# Aktuelle Therapien, absehbare Newcomer und neue Therapieansätze für die Sjögren-Erkrankung

**DOI:** 10.1007/s00393-026-01815-3

**Published:** 2026-04-27

**Authors:** Diana Ernst, Nadine Zehrfeld

**Affiliations:** Klinik für Rheumatologie & Immunologie, Medizinische Hochschule Hannover, Carl-Neuberg-Str. 1, 30625 Hannover, Deutschland

**Keywords:** Sjögren-Syndrom, Biologika, Sicca-Symptomatik, Ianalumab, Teletacicept, Sjögren’s syndrome, Biologicals, Ianalumab, Sicca syndrome, Teletacicept

## Abstract

Obwohl die Sjögren-Erkrankung (SjD) die häufigste Kollagenose ist, verfügen wir bislang über keine zugelassenen Medikamente für die systemische Behandlung. Viele DMARDs („disease-modifying antirheumatic drugs“) und Biologika konnten in Studien keine ausreichende Wirksamkeit zeigen. Gründe hierfür sind u. a. komplexe immunologische Pathomechanismen sowie eine hohe klinische und immunologische Heterogenität der Patient:innen. In den letzten Jahren kam es jedoch zu deutlichen Fortschritten: Erstmals berichteten Phase-3-Studien 2025 (Ianalumab und Telitacicept) positive Ergebnisse. Grundlage der aktuellen Therapieempfehlungen sind v. a. die Leitlinien der European Alliance of Associations for Rheumatology (EULAR, 2020) und die Empfehlungen der British Society for Rheumatology (BSR, 2024). Die Therapie orientiert sich aktuell primär an den führenden Symptomen. Basis ist die konsequente Behandlung der Sicca-Symptomatik (Augen- und Mundpflege, ggf. Pilocarpin). Extraglanduläre Manifestationen werden überwiegend off-label mit konventionellen DMARDs behandelt; bei schweren Verläufen kommen Rituximab, seltener Cyclophosphamid oder intravenöse Immunoglobuline (IVIG) zum Einsatz. Neue Therapieansätze zeigen erstmals eine anhaltende Reduktion der Krankheitsaktivität: Besonders B‑Zell-gerichtete Therapien (Ianalumab, Telitacicept), FcRn-Inhibitoren (Nipocalimab, Efgartigimod), CD40/CD40L-Blockade, Kinase-Inhibitoren und weitere immunmodulatorische Strategien erzielten in kontrollierten Studien signifikante Verbesserungen im ESSDAI (EULAR Sjögren’s Syndrome Disease Activity Index) und teils im ESSPRI (EULAR Sjögren’s Syndrome Patient-Reported Index) bei akzeptablem Sicherheitsprofil. Während Basismaßnahmen unverzichtbar bleiben, markieren neue zielgerichtete Therapien einen Wendepunkt in der SjD-Behandlung. Insbesondere B‑Zell- und FcRn-gerichtete Ansätze eröffnen erstmals die Perspektive auf evidenzbasierte systemische Therapien über die reine Symptomkontrolle hinaus.

## Abstract (en)

Although Sjögren’s disease (SjD) is the most frequent connective tissue disease, there are still no approved medications for systemic treatment. Many disease-modifying antirheumatic drugs (DMARD) and biologicals have not shown sufficient efficacy in studies. The reasons for this are the complex immunological mechanisms and a high degree of clinical and immunological heterogeneity of the patients; however, significant progress has been made in recent years: for the first time 2 phase 3 studies (ianalumab and telitacicept) reported positive results. The current treatment recommendations are based on the European Alliance of Associations for Rheumatism (EULAR) guidelines (2020) and the recommendations of the British Society for Rheumatology (2024). The treatment is currently primarily based on the leading symptoms and includes consistent treatment of dry eye and dry mouth symptoms (eye and mouth care, pilocarpine if necessary). Extraglandular manifestations continue to be treated predominantly off-label with conventional DMARDs; in severe courses rituximab is used and less frequently cyclophosphamide or intravenous immunoglobulins (IVIG). New treatment approaches show consistent effects on reduction of disease activity (EULAR Sjogren’s syndrome disease activity index, ESSDAI) for the first time: In particular, B‑cell-targeted therapies (ianalumab, telitacicept), FcRn inhibitors (nipocalimab, efgartigimod), CD40/CD40L blockade, kinase inhibitors and other immunomodulatory strategies have achieved significant improvements in ESSDAI and, in some cases EULAR Sjögren’s syndrome patient reported index (ESSPRI) with an acceptable safety profile. Although basic measures remain indispensable, new forms of targeted treatment mark a turning point in the treatment of SjD. In particular, B‑cell and FcRn-targeted approaches open up for the first time the prospect of evidence-based systemic treatment that goes beyond mere symptom control.

Bis vor wenigen Jahren gab es kaum positive Ergebnisse bei Therapiestudien zur Sjögren-Erkrankung (SjD). Eine unzureichende Wirkung vieler neuerer Wirkstoffe wurde berichtet (TNF-Inhibitoren, Abatacept, Rituximab) [1–8]. Hauptursachen hierfür sind die komplexen immunologischen Mechanismen der Erkrankung sowie die ausgeprägte klinische und immunologische Heterogenität der Patient:innenkollektive, doch auch ungünstig gewählte Endpunkte spielten dabei eine Rolle [9]. Aufgrund der deutlichen klinischen Heterogenität der SjD-Kohorten fanden in den letzten Jahren diverse Cluster-Analysen statt, um den klinischen Phänotyp besser einordnen und verstehen zu können. So konnte z. B. auch durch die transkriptomische Stratifizierung unterschiedliches Therapieansprechen auf Hydroxychloroquin/Leflunomid und Rituximab bei SjD-Patient:innen gesehen werden. Patient:innen mit einer geringen Krankheitsaktivität und dem entsprechendem transkriptomischen Profil zeigten hingegen kein relevantes Ansprechen. Aktuell gibt es zusätzlich diverse Ansätze und Entwicklungen, Biomarker zu finden, die bei Therapieentscheidungen helfen können [10].

Internationale Handlungsempfehlungen werden derzeit vor allem durch die Leitlinien der European Alliance of Associations for Rheumatology (EULAR) von 2020 sowie die Empfehlungen der British Society for Rheumatology (BSR) aus dem Jahr 2024 abgedeckt [2, 11].

Die Therapie der Sicca-Symptomatik stellt eine Basistherapie dar

Die Therapie der SjD orientiert sich dabei v. a. an den klinisch führenden Symptomen. Entsprechend stellt die Therapie der mehrheitlich vorhandenen Sicca-Symptomatik eine Art Basistherapie dar. Diese sollte zum Schutz vor Karies und Parodontitis sowie okulären Spätfolgen, wie Ulzerationen und andere Schäden an der Cornea, auch konsequent durchgeführt werden.

Bei extraglandulärem Befall bewegen wir uns immer noch therapeutisch im Off-label-Bereich. Die Therapieempfehlungen orientieren sich unter anderem, je nach Manifestation und Klinik, an den Therapieempfehlungen des systemischen Lupus erythematodes (SLE), der Kleingefäßvaskulitiden oder, besonders bei neurologischer Manifestation, der multiplen Sklerose [2, 11]. Erstmalig konnten letztes Jahr Phase-3-Studien positive Ergebnisse berichten (*Ianalumab* und *Teletacicept*), und weitere Phase-3-Studien, mit positiven Phase-2-Studien, werden aktuell durchgeführt.

## Therapie der Sicca-Symptomatik

Sowohl die EULAR-Empfehlungen von 2020 als auch die Empfehlungen der BSR von 2024 empfehlen diesbezüglich wichtige Basismaßnahmen, die bei jeder Diagnosebesprechung mit den Patient:innen erwähnt und auch schriftlich mitgegeben werden sollten [2, 11]. Tab. 1 fasst die wichtigsten Maßnahmen zur Sicca-Therapie zusammen. Eine sorgfältige Mundhygiene sowie zahnärztliche Reinigungen zur Kariesprophylaxe sind dabei ebenso wichtig wie eine symptomorientierte Steigerung der Augentropfen-Therapie, von rein befeuchtenden Augentropfen bis hin zu serumbasierten Augentropfen, dem Einsatz von topischen Glukokortikoiden oder Ciclosporin-haltigen Augentropfen. Speichelersatzprodukte finden oft keinen dauerhaften Einsatz, da sie den Patient:innen kaum Vorteile bieten, zumeist teuer sind und zudem den Geschmack der Patient:innen oftmals nicht treffen. Entsprechend werden eher eine Flasche Wasser, zuckerfreie saure Drops, Lutschtabletten oder Kaugummis gewählt.

**Tab. 1:** Übersicht zu den therapeutischen Empfehlungen der EULAR (2020) und der BSR (2024) zur Behandlung der okkulären und oralen Sicca-Symptomatik

	Augentrockenheit	Mundtrockenheit
Leichtgradig	Befeuchtende Augentropfen Lidkompressen	Saure Lutschtabletten, Wassertrinken, zuckerfreie Kaugummis Sorgfältige Zahnpflege und Mundhygiene Fluoridhaltige Kariesprophylaxen
Mittelgradig	Künstliche Tränen/pflegende Gele Kurzfristig topische NSAR/Glukokortikoide	Elektrostimulation der Speicheldrüsen Speichelersatzmittel
Schwergradig	Topisches Ciclosporin A Serum-Augentropfen In ausgewählten Fällen: Tränenkanalverschluss (Punktum-Plugs) Systemische Sekretomimetika	Systemische Sekretomimetika: Pilocarpin

Anmerkung: Unter „leichtgradig“ aufgeführte Maßnahmen verstehen sich Basistherapien, zu denen gemäß Bedarf die Therapie eskaliert werden kann

*EULAR* European Alliance of Associations for Rheumatology, *BSR* Britische Gesellschaft für Rheumatologie, *NSAR* nichtsteroidale Antirheumatika

Auch der Einsatz von *Pilocarpin* bei systemischer Sicca-Symptomatik wird nur von einem kleinen Teil der Patient:innen toleriert, da Kopfschmerzen, Flush-Symptomatik, Schwindel oder auch fehlende subjektive Besserung einen dauerhaften Einsatz oftmals verhindern. Einen Therapieversuch mit *Pilocarpin* zu versuchen, ist bei gegebener Indikation dennoch zu empfehlen, da ein (geringer) Teil der Patien:innen durchaus sehr davon profitiert. Sowohl *Pilocarpin* als auch Cyclosporin-haltige Augentropfen sind für die SjD zugelassen und werden von den gesetzlichen Krankenkassen übernommen. Gleiches gilt auch für einige weitere Augentropfen [12].

Wenig Beachtung in den aktuellen Empfehlungen finden aktuell Maßnahmen zur Therapie bei vaginaler Sicca-Symptomatik, Rhinitis sicca oder auch ausgeprägter Xerodermie. Bei der vaginalen Sicca-Symptomatik, die bei bis zu 60 % der Patientinnen auftritt, sind Hyaluronsäure und/oder lipidhaltige Gele und Gleitmittel empfohlen. Gegebenenfalls kommen auch hormonelle Therapien in Frage.

Bekannte Biologika, wie *Abatacept, Belimumab* und auch *Rituximab* zeigten zum Teil positive Effekte auf den Speichelfluss, finden aber für diese Indikation keinen Einsatz in der klinischen Praxis [13].

Nipocalimab und Iscalimab konnten in Phase-2-Studien den Speichelfluss verbessern

**Abb. 1:**
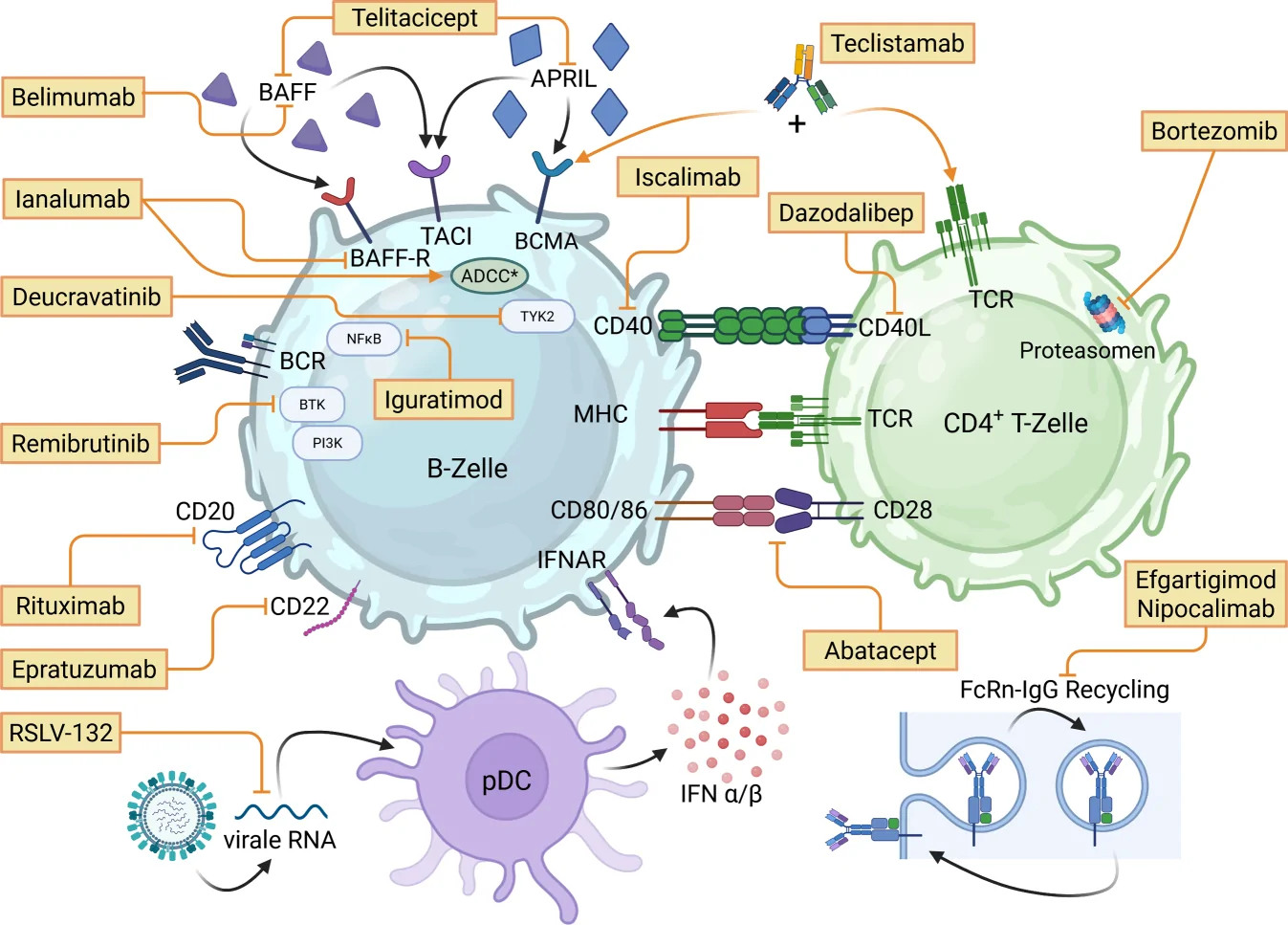
Übersicht zu den Wirkprinzipien in Erprobung befindlicher und etablierter Therapien der Sjögren-Erkrankung. *L* Ligand, *R* Rezeptor, *pDC* periphere dendritische Zelle, *CD* Cluster of differentiation,* MHC* Major Histokompatibility Komplex, *TCR* T-Zellrezeptor (CD3),* BCR* B-Zell Rezeptor, *TYK2* Tyrosinkinase 2, *BTK* Bruton Tyrosinkinase, *NFκB* nuclear factor ‚kappa-light-chain-enhancer‘ of activated B‑cells, *PI3K* Phosphoinosid-3-Kinase, *BCMA* B-Zell Maturation Antigen,* TACI* Transmembrane Activator and CAML-interactor, *BAFF* B-Zell aktivierender Faktor, *APRIL* A proliferation-inducing ligand,* IFN* Interferon, *IFNAR* Interferon A Rezeptor,* FcRN* neonataler Fc-Rezeptor,* IgG* Immunglobulin G, ADCC* antikörperabhängige zelluläre Toxizität. (Created with BioRender Zehrfeld N. (2026) https://BioRender.com/hql1bad)

In aktuellen Studien konnten auch neue Wirkstoffe, deren Wirkmechanismen im nachfolgenden Teil beschrieben werden und in Abb. 1 dargestellt sind, einen positiven Effekt auf die Speicheldrüsenfunktion zeigen. So konnten *Nipocalimab* und *Iscalimab* in Phase-2-Studien den Speichelfluss verbessern [14, 15]. Gleiches gilt für *Ianalumab*, der Wirkstoff, der nach positiver Phase-3-Studie gute Chancen auf eine zeitnahe Zulassung hat [16].

Frühe Entwicklungsphasen gibt es zudem hinsichtlich des Einsatzes von mesenchymalen Stromazellen und stammzellbasierten Therapieansätzen aus dem Fettgewebe sowie gentherapeutischen Ansätzen in sehr frühen Entwicklungsphasen, welche aktuell weiterverfolgt werden [17, 18].

## Therapie der extraglandulären Manifestationen

Obwohl bislang keine zugelassene systemische Therapie für die SjD existiert, werden verschiedene systemisch wirksame immunsuppressive Medikamente eingesetzt. Die Auswahl der Therapie richtet sich dabei primär nach dem klinischen Erscheinungsbild und der Schwere der Organbeteiligung und folgt in der klinischen Praxis häufig therapeutischen Prinzipien, die aus der Behandlung des SLE bekannt sind. Mit der Veröffentlichung der EULAR-Empfehlungen im Jahr 2020 und den Empfehlungen der BSR 2024 stehen differenzierte, indikationsspezifische Behandlungsalgorithmen zur Verfügung, die je nach führender Manifestation unterschiedliche therapeutische Vorgehensweisen vorschlagen [2, 11], wobei die Empfehlungen der BSR insbesondere auch nichtmedikamentöse Ansätze, wie Bewegungstherapie, bei im Vordergrund stehenden Schmerzen oder Fatigue aufführt.

Hydroxychloroquin kann zur Kontrolle von entzündlichen Gelenk- und Muskelschmerzen verwendet werden

Bei überwiegend muskuloskeletaler Symptomatik kommen klassische krankheitsmodifizierende Antirheumatika zum Einsatz. *Hydroxychloroquin (HCQ)* wird insbesondere zur Kontrolle von Gelenk- und Muskelschmerzen verwendet, dafür zeigte es auch einen signifikanten Effekt bei Patient:innen mit SjD. Während keine Verbesserung der Sicca-Symptomatik beobachtet werden konnte, kam es zusätzlich zu einer signifikanten Reduktion der Blutsenkungsgeschwindigkeit unter HCQ [19].

*Methotrexat* oder *Azathioprin* werden dagegen bevorzugt bei entzündlicher Arthritis sowie bei mild ausgeprägten kutanen Manifestationen eingesetzt. Aktuellere Daten aus Phase-2a- und -2b-Studien gibt es auch zur Kombinationstherapie von Leflunomid und HCQ [20]. Für eine relativ kleine Fallzahl konnte eine signifikante Reduktion des ESSDAI (EULAR Sjögren’s Syndrome Disease Activity Index) gezeigt werden, auch die zusammensetzten Endpunkte CRESS (Composite of Relevant Endpoints for Sjögren’s Syndrome) und STAR (Sjögren’s Tool for Assessing Response) wurden erreicht [21, 22]. Doch auch in der Kombination HCQ mit Leflunomid wurde keine Verbesserung der Sicca-Symptomatik gemessen. Entwickelt sich ein schwerer Krankheitsverlauf mit unzureichendem Ansprechen auf konventionelle Therapien oder kommt es zu einer relevanten Organbeteiligung, wird von den EULAR-Expert:innen der Einsatz von B‑Zell-depletierenden Therapien, insbesondere *Rituximab*, empfohlen. Eine aktuelle Metaanalyse zur Rituximab-Therapie konnte nur in der gepoolten Auswertung einen signifikanten Effekt zur Reduktion der Krankheitsaktivität finden. Nur 4/14 Studien mit Rituximab konnten zeigen, dass sich die Sicca-Symptomatik verbesserte. Die beste Evidenz für Rituximab gibt es für vaskulitische Manifestationen [23]. Alternativ hierzu kann *Cyclophosphamid* erwogen werden, das vor allem bei vaskulitischer Beteiligung des zentralen Nervensystems, ausgeprägten Polyneuropathien sowie bei refraktären pulmonalen oder renalen Manifestationen noch eine zentrale Rolle spielt. Zur Behandlung peripherer Nervenschädigungen haben sich regelmäßige intravenöse *Immunglobulingaben* als effektive therapeutische Option etabliert.

Im Vergleich zu den EULAR-Empfehlungen empfiehlt die BSR auch den Einsatz von *Colchizin* bei vaskulitischen Manifestation sowie bei Panniculitis und Perikarditis im Rahmen einer SjD [2].

Derzeit werden in den EULAR-Empfehlungen lediglich drei Antikörpertherapien explizit berücksichtigt: *Abatacept, Belimumab* und *Rituximab*. Der Einsatz von *Belimumab* ist dabei auf spezielle refraktäre Manifestationen begrenzt und wird insbesondere bei persistierenden Parotisschwellungen in Erwägung gezogen.

Unabhängig von den genannten Therapieempfehlungen publizierte eine portugiesische Arbeitsgruppe im Jahr 2023 ergänzende Empfehlungen zum gezielten Einsatz biologischer Therapien [24]. Für Patient:innen mit isolierter Drüsentrockenheit oder vorherrschender Fatigue wird darin weiterhin von einer biologischen Therapie abgeraten. Demgegenüber wird bei ausgeprägter systemischer Krankheitsaktivität in Kombination mit einem erhöhten Risiko für die Entwicklung eines Lymphoms ein frühzeitiger Beginn einer *Rituximab*-Therapie befürwortet. Zeigt *Rituximab* allein keine ausreichende Wirksamkeit, wird die Kombination mit *Belimumab* als mögliche Eskalationsstrategie vorgeschlagen.

Bei einer Therapie mit *Abatacept* konnten in offenen klinischen Studien zwar positive Effekte auf Fatigue, Lebensqualität und den ESSDAI-Score gesehen werden, diese Ergebnisse ließen sich jedoch in kontrollierten Phase-3-Studien nicht bestätigen. Vor diesem Hintergrund wird *Abatacept* in den EULAR-Empfehlungen von 2020 lediglich noch als Reserveoption bei therapierefraktärer Arthritis aufgeführt [1, 25].

### Neue Wirkstoffe auf dem Vormarsch

In den letzten Jahren hat sich ein breites Spektrum neuer immunmodulatorischer Therapien in der klinischen Entwicklung etabliert. Die Identifikation zahlreicher Angriffspunkte spiegelt die Vielschichtigkeit der Krankheitsentstehung wider (Abb. 1; Tab. 2). Zu den zentralen Strategien zählen die gezielte Beeinflussung der B‑Zell-Homöostase über BAFF- und APRIL-Signalwege, die Unterbrechung kostimulatorischer Interaktionen zwischen Immunzellen, die Hemmung intrazellulärer Signaltransduktionskaskaden sowie die Reduktion pathogener Autoantikörper durch Blockade des neonatalen Fc-Rezeptors. Kombinationstherapien, insbesondere die sequenzielle Anwendung von B‑Zell-depletierenden und B‑Zell-modulierenden Substanzen, scheinen eine intensivere und länger anhaltende Immunmodulation zu ermöglichen.

**Tab. 2:** Übersicht zu den molekularen Zielstrukturen der erwähnten zielgerichteten Wirkstoffe und aktueller Studienstand

Wirkstoff	Molekulare Zielstruktur	Studien abgeschlossen
*Rituximab*	CD20	**Phase 3**; *primärer Endpunkt in Bezug auf Speicheldrüsenfunktion erreicht *[8, 39] und *primärer Endpunkt nicht erreicht in Bezug auf systemisches Ansprechen *[40]
*Belimumab*	BAFF	**Phase 2** für Kombi mit Rituximab; *primärer Endpunkt erreicht in Bezug auf Speicheldrüsenfunktion* [29] **Beobachtungsstudien** [28, 41, 42]
*Ianalumab*	BAFF-R/ADCC	**Phase 3**; *primärer Endpunkt in Bezug auf klinisches Ansprechen erreicht* [4]
*Telitacicept*	BAFF und APRIL	**Phase 3**; *primärer Endpunkt in Bezug auf klinisches. Ansprechen erreicht* [26]
*Epratuzumab*	CD22	**Phase 2**; *primärer Endpunkt in Bezug auf klinisches Ansprechen erreicht *[30]; *(Phase 3 in SLE hat primäre Endpunkte in Bezug auf klinisches Ansprechen erreicht)* [43]
*Abatacept*	CD80/86	**Phase 3**; *primärer Endpunkt in Bezug auf klinisches Ansprechen nicht erreicht* [1]
*Iscalimab*	CD40	**Phase 2***; primärer Endpunkt in Bezug auf klinisches Ansprechen erreicht *[3]*; aufgrund von Sicherheitsproblemen in Studien anderer Indikationen Weiterentwicklung eingestellt*
*Dazodalibep*	CD40‑L	**Phase 2**; *primärer Endpunkt in Bezug auf klinisches Ansprechen erreicht *[31]*. Phase 3 für SjD läuft*
*Bortezomib*	26-S-Proteasom	**Fallberichte** [44]
*RSLV-132*	RNA-Moleküle an Auto-AK	**Phase 2**; *primärer Endpunkt in Bezug auf klinisches Ansprechen erreicht *[45]
*Iguratimod*	NF-κB-Signalweg	**Phase 2**; *primärer Endpunkt in Bezug auf klinisches Ansprechen erreicht* [46]
*Nipocalimab*	FcRn	**Phase 2**; *primärer Endpunkt in Bezug auf klinisches Ansprechen erreicht* [14]. *Phase 3 für SjD läuft*
*Efgartigimod*	FcRn	**Phase 2**; *primärer Endpunkt in Bezug auf klinisches Ansprechen erreicht* [32]. *Phase 3 für SjD läuft*
*Deucravacitinib*	TYK2	*(****Phase 2 bei SLE*** *hat; primärer Endpunkt in Bezug auf klinisches Ansprechen erreicht)* [33]. *Phase 3 für SjD läuft*
*Remibrutinib*	BTK	**Phase 2**; *primärer Endpunkt in Bezug auf klinisches Ansprechen erreicht* [47]
*Teclistamab*	CD3/BCMA	**Fallberichte** [34]

*R* Rezeptor, *L* Ligand, *CD* Cluster of Differentiation, *RNA* Ribonucleinsäure, *BAFF* B-Zell aktivierender Faktor, *APRIL* A-Proliferation-Inducing Ligand, *Auto-AK* Autoantikörper, *TYK2* Tyrosinkinase 2, *BTK* Bruton-Tyrosinkinase, *BCMA* B-cell maturation antigen, *FcRN* neonataler Fc Rezeptor, NF*κB* nuclear factor ‚kappa-light-chain-enhancer‘ of activated B‑cells

Während zahlreiche Substanzen aufgrund mangelnder Wirksamkeit oder unerwünschter Nebenwirkungen nicht weiterverfolgt wurden, erzielten einzelne zielgerichtete Therapien in frühen Studien klinisch relevante Effekte. Wirkstoffe wie *Belimumab, Ianalumab, Iscalimab, Dazodalibep, Remibrutinib, Telitacicept* und *Nipocalimab* erreichten in kontrollierten Studien ihre primären Endpunkte [3, 8, 14, 16, 26, 27].

### B-Zell-gerichtete Therapien

*Belimumab* zeigte in einer placebokontrollierten Studie (BLISS) eine nachhaltige Reduktion der systemischen Krankheitsaktivität (ESSDAI) und der subjektiven Symptomlast (EULAR Sjögren’s Syndrome Patient-Reported Index, ESSPRI) nach 52 Wochen im Vergleich zur Baseline [28]. In Kombination mit *Rituximab *konnte zudem eine ausgeprägte B‑Zell-Depletion im peripheren Blut und in den Speicheldrüsen nachgewiesen werden.

Das Rennen um die erste Zulassung zur systemischen Therapie bei extraglandulärer Manifestation der SjD für neue Therapieansätze ist längst eröffnet, und aktuell hat *Ianalumab* die Nase vorn [29].

*Ianalumab* ist ein humaner monoklonaler Antikörper, der sowohl zur B‑Zell-Depletion als auch zur Blockade des BAFF-Rezeptor führt und somit die B‑Zell-Funktion über zwei Ansätze inhibiert. Das Erreichen der Endpunkte der Phase-3-Studien wurde bereits im August 2025 verkündet und auf dem ACR 2025 in Chicago die ersten Daten dazu präsentiert. Da *Ianalumab* aktuell der Wirkstoff ist, der einer Zulassung am nächsten scheint, werden die Studiendaten im Folgenden vorgestellt.

Das Rennen um die erste Zulassung zur systemischen Therapie ist längst eröffnet

Die Wirksamkeit und Verträglichkeit von *Ianalumab* wurde in zwei groß angelegten Phase-3-Studien untersucht [4]. In die Studie NEPTUNUS‑1 wurden insgesamt 275 erwachsene Patient:innen eingeschlossen und im Verhältnis 1:1 randomisiert, während NEPTUNUS‑2 504 Teilnehmende umfasste, die im Verhältnis 1:1:1 zugeteilt wurden. Beide Studien hatten eine Laufzeit von 52 Wochen. Voraussetzung für die Studienteilnahme war eine systemische Krankheitsaktivität mit einem ESSDAI-Score von mindestens 5 in 8 vordefinierten Organdomänen sowie eine stimulierte Speichelflussrate von mindestens 0,05 ml/min. Sowohl NEPTUNUS‑1 als auch NEPTUNUS‑2 wurden als multizentrische, randomisierte, doppelblinde und placebokontrollierte Studien konzipiert. Eine stabile Begleittherapie war in beiden Studien zulässig und konnte *Methotrexat* (bis zu 25 mg/Woche), *Hydroxychloroquin* (bis zu 400 mg/Tag), *Azathioprin* (bis zu 150 mg/Tag) sowie *Prednison* (bis zu 10 mg/Tag) umfassen, entweder als Monotherapie oder in Kombination. Im Rahmen von NEPTUNUS‑1 erhielten die Teilnehmenden entweder 300 mg *Ianalumab* oder Placebo in monatlichen Abständen. In NEPTUNUS‑2 wurden 3 Behandlungsarme untersucht: eine monatliche Gabe von 300 mg *Ianalumab*, eine vierteljährliche Gabe derselben Dosis oder Placebo. Beide Studien erreichten ihren primären Endpunkt, der in der Überlegenheit gegenüber Placebo hinsichtlich der Veränderung des ESSDAI vom Ausgangswert bis Woche 48 definiert war. Insbesondere die monatliche Applikation von *Ianalumab* erwies sich als statistisch signifikant wirksamer als Placebo, mit einer stärkeren Reduktion des ESSDAI (−6,6 vs. −5,5). Auch bei mehreren sekundären Endpunkten, darunter weitere Parameter der Krankheitsaktivität, zeigte sich in beiden Studien eine konsistente Tendenz zugunsten von *Ianalumab*. Die Sicherheitsbewertung erfolgte über den gesamten Studienzeitraum von 52 Wochen. Dabei zeigte *Ianalumab* ein insgesamt günstiges Sicherheitsprofil, wobei Häufigkeit und Schwere unerwünschter sowie schwerwiegender unerwünschter Ereignisse mit denen der Placebogruppe vergleichbar waren. Zu erwähnen ist noch ein relativ hohes Ansprechen der Placebogruppe, ähnlich wie wir es aus vielen SLE-Studien kennen, mit einer ESSDAI-Reduktion bei 89 % (300 mg Ianalumab) vs. 61 % (Placebo).

Nur kurz nach der Verkündigung der positiven Phase-3-Studie von *Ianalumab* wurden die ebenfalls positiven Daten von *Telitacicept* bekanntgegeben. *Telitacicept* ist ein rekombinantes Fusionsprotein, das sich sowohl gegen den B‑Zell-aktivierenden Faktor (BAFF) als auch gegen den A‑Proliferation-Inducing Ligand (APRIL) richtet. Beide Moleküle gehören zur Tumornekrosefaktor-Familie, können als Schwesterzytokine angesehen werden und haben überlappende Zielrezeptoren. Beide Zytokine sind zentrale Mediatoren für die Reifung, das Überleben und die Aktivierung von B‑Zellen, die bei autoimmunen Prozessen wie der SjD eine zentrale Rolle spielen. Durch diese duale Hemmung wird die pathologisch gesteigerte B‑Zell-Antwort reduziert. Unter der Behandlung mit *Telitacicept* zeigte sich über einen Zeitraum von 24 Wochen eine klinisch relevante, dosisabhängige Abnahme sowohl der systemischen Krankheitsaktivität als auch der subjektiven Symptomlast, gemessen anhand der ESSDAI- und ESSPRI-Scores [26]. Die Phase-2b-Studie wurde ausschließlich in China durchgeführt und zeigte ein auffallend niedriges Placeboansprechen, sodass die Daten der aktuell international laufenden Phase-3-Studie von *Telitacicept* in voraussichtlich 2027 mit Spannung erwartet werden.

Doch auch andere B‑Zell-gerichtete Therapien wie *Epratuzumab*, ein Antikörper gegen CD22, zeigte eine signifikante Verbesserung der Fatigue bei SjD-Patient:innen und wird aktuell noch weiter verfolgt [30].

### CD40/CD40L-Kostimulation

Die Blockade der CD40/CD40L-Achse erwies sich insbesondere bei Patient:innen mit höherer systemischer Aktivität als wirksam.

*Iscalimab* und *Dazodalibep* greifen gezielt in die Aktivierung von T‑Zellen ein, indem sie die kostimulatorische Signalübertragung über CD40 bzw. dessen Liganden CD40L unterbrechen [27, 31]. Dieser therapeutische Ansatz ist insbesondere vor dem Hintergrund der frühen Krankheitsstadien der SjD plausibel: Histopathologische Untersuchungen zeigen, dass initial vor allem CD4⁺-T-Zellen die entzündlichen Infiltrate der Speicheldrüsen dominieren, während B‑Zellen typischerweise erst im weiteren Krankheitsverlauf eine zunehmende Rolle spielen. Die Blockade der T‑Zell-Kostimulation adressiert somit einen zentralen Mechanismus der frühen Immunaktivierung.

Eine randomisierte klinische Studie zur Wirksamkeit von *Dazodalibep* zeigte, dass bei Patient:innen mit moderater bis schwerer systemischer Krankheitsaktivität nach 24 Wochen eine signifikante Reduktion des ESSDAI erreicht werden konnte. Eine signifikante Verbesserung des ESSPRI wurde hingegen vor allem bei Betroffenen mit ausgeprägter subjektiver Symptomatik, jedoch nur geringer Organbeteiligung, beobachtet [27].

*Iscalimab* zeigte in der TWINSS-Studie dosisabhängige signifikante Effekte bezüglich einer ESSDAI-Reduktion und war hinsichtlich der Sicherheitsdaten vergleichbar mit Placebo [15]. Aufgrund von Sicherheitsdaten aus anderen Indikationen entschied sich der Hersteller allerdings, den Wirkstoff nicht länger zu untersuchen.

### FcRn-Inhibitoren

*Nipocalimab* und *Efgartigimod*, beides FcRn-Inhibitoren, reduzieren sowohl die Krankheitsaktivität als auch zirkulierende Autoantikörper, was den postulierten Wirkmechanismus bestätigt. *Nipocalimab* erfüllte in der DAHLIAS-Studie den primären Studienendpunkt, der in einer signifikanten Reduktion des klinisch erhobenen ESSDAI nach 24 Wochen bestand. Parallel hierzu zeigte sich eine deutliche Abnahme zirkulierender Autoantikörper, was den postulierten Wirkmechanismus des Antikörpers widerspiegelt [14]. Diese Daten und die bereits bestehende Zulassung des Wirkstoffs bei Myasthenia gravis führten zu einem bevorzugten „Fast-Track-Status“ für eine mögliche Zulassung der FDA (US Food and Drug Administration).

*Efgartigimod* zeigte in der Phase-2-Studie RHO im Vergleich zu Placebo Verbesserungen bei den klinischen Endpunkten (inkl. ESSDAI) sowie eine deutliche Reduktion von Autoantikörpern wie anti-Ro52 und Rheumafaktor. Die Behandlung war gut verträglich [32].

Auf Grundlage dieser Ergebnisse wurde 2025 die Phase-3-Studie UNITY initiiert, eine multizentrische, randomisierte, doppelblinde, placebokontrollierte Untersuchung über 48 Wochen, um Wirksamkeit und Sicherheit bei Patient:innen mit moderater bis schwerer SjD zu evaluieren. *Efgartigimod* ist in Europa und der Schweiz ebenfalls für die Myasthenia gravis bereits zugelassen.

### Kinase-Inhibitoren

Weitere Wirkstoffe, die in fortgeschrittenen Entwicklungsphasen untersucht werden, umfassen *Deucravacitinib*, ein TYK-2-Inhibitor, und *Remibrutinib,* ein BTK-Inhibitor.

*Deucravacitinib* zeigte in der SLE-Studie PAISLEY eine besonders gute Wirkung für Schmerz und Fatigue-Symptomatik bei Ro-Antikörper positiven SLE-Patient:innen, sodass diese Daten als Grundlage dienten, unmittelbar eine Phase-3-Studie für die Effektivität bei der SjD zu initiieren [33].

*Remibrutinib* reduzierte in einer Phase-2-Studie bei Patient:innen mit SjD über 24 Wochen die systemische Krankheitsaktivität deutlich im Vergleich zu Placebo, während keine Wirkung auf die patient:innenberichteten Symptome (ESSPRI) beobachtet wurde. Es zeigte sich zudem ein Trend zur Verbesserung des unstimulierten Speichelflusses.

### Proteasomeninhibitor, Blockade des NF-κB-Signalwegs, RNase-Fc-Fusionsprotein RSLV-132 und andere

Bortezomib, ein Proteasomeninhibitor, der B‑Zellen und Plasmazellen gezielt zum Absterben bringt, konnte in einzelnen Fallberichten bei therapierefraktären Patient:innen zu einer deutlichen Verbesserung von Fatigue, Sicca-Symptomen und systemischer Krankheitsaktivität führen.

*Iguratimod* wirkt als immunmodulatorische Substanz, indem es mehrere entzündliche Signalwege gleichzeitig beeinflusst. Es hemmt die Produktion proinflammatorischer Zytokine wie IL‑6, IL-17 und TNF‑α, blockiert die Aktivierung des NF-κB-Signalwegs und reduziert die Antikörpersekretion durch B‑Zellen. Es konnte gezeigt werden, dass *Iguratimod* die Fatigue und den ESSPRI-Score signifikant bei SjD-Patient:innen verbessern konnte. Diese Daten stammen bislang aus Fallserien oder frühen klinischen Studien und zeigen, dass neue therapeutische Strategien bei refraktären oder schwer betroffenen Patient:innen mit SjD Potenzial besitzen, sie müssen jedoch in größeren kontrollierten Studien noch validiert werden.

*RSLV-132* ist ein rekombinantes Fusionsprotein, das aus einer enzymatisch aktiven humanen Ribonuklease (RNase) und der Fc-Domäne eines humanen IgG1-Antikörpers besteht. Dadurch wird eine verlängerte Halbwertszeit erreicht und der gezielte Abbau extrazellulärer RNA ermöglicht. In einer randomisierten klinischen Studie konnte eine signifikante Verbesserung von Fatigue bei Patient:innen mit SjD gesehen werden. Aktuell wird eine globale Phase-2-Studie für SjD-Patien:innen durchgeführt, Ergebnisse werden im Jahr 2027 erwartet. Bezüglich Fatigue laufen aktuell auch Studien für den Wirkstoff bei SLE und Long-COVID.

Andere neue, noch sehr frühe, Therapieansätze (Fallberichte) schließen auch bispezifische T‑Zell-Engager-Therapien mit z. B: *Teclistamab* (CD3/BCMA) ein [34]. Fallserien zum Einsatz von T‑Zell-Engager-Therapien liegen derzeit zu therapierefraktärem SLE, Rheumatoider Arthritis, Systemischer Sklerose und Antisynthethase-Syndrom vor [35–38].

## Fazit für die Praxis

- Es bewegt sich aktuell viel in der Entwicklung neuer Therapieoptionen bei der Sjögren-Erkrankung (SjD).
- Je besser wir die Pathomechanismen verstehen, um so gezielter können Therapieoptionen getestet und weiterentwickelt werden.
- Basismaßnahmen wie die Behandlung der Sicca-Symptomatik bleiben zentral, während extraglanduläre Manifestationen aktuell off-label mit bekannten DMARDs („disease-modifying anti-rheumatic drugs“) behandelt werden.
- Nur eine geringe Auswahl an Biologika wird aktuell bei therapierefraktären Verläufen oder vaskulitischen Manifestationen empfohlen.
- Die neuen Therapieansätze umfassen B‑Zell-gerichtete Therapien, FcRn-Inhibitoren, CD40/CD40L-Blockade, Kinase-Inhibitoren sowie proteasomenbasierte oder NF-κB-modulierende Strategien.
- Insgesamt eröffnen diese Entwicklungen, insbesondere die Daten zu den B‑Zell- und FcRn-gerichteten Therapien, erstmals die Aussicht auf gezielte, evidenzbasierte Therapien über die reine Symptomkontrolle hinaus.
